# A Low-Cost Task Trainer Constructed from Silicone Nipple Covers

**DOI:** 10.21980/J8.52244

**Published:** 2025-10-31

**Authors:** Aubrey Bethel, Vivienne Ng

**Affiliations:** * Creighton University East Valley, Department of Emergency Medicine, Phoenix, AZ; ^ The University of Arizona College of Medicine Tucson, Department of Emergency Medicine, Tucson, AZ

## Abstract

**Audience:**

This low-cost task trainer is intended for the education of medical students, advanced practice providers and surgical subspecialties interns, including emergency medicine.

**Introduction:**

Superficial soft-tissue abscesses are a frequent chief complaint in any emergency department, with up to 3.2 % of patients presenting with this issue.^1^ The preferred method for treatment is incision and drainage (I&D) because antibiotics alone are often insufficient.^2^,^3^ There are two common methods for draining abscesses. The first is a single linear incision over the length of the abscess that is either left open or packed with gauze which is removed 24–48 hours later.^4^ The second is the loop technique, which uses two smaller parallel incisions with a sterile rubber or plastic tube threaded through them and tied into a circle.^5^,^6^

While abscess drainage is a common procedure for surgical and sub-surgical specialties, it is not often taught in medical schools or to residency prior to performing in the patient care setting. Frequently, this is due to the to lack of access to affordable commercial task trainers, which range in cost from $19.99 up to $171.00 per single use device.^7^,^8^ Other published low-cost task trainers require cadavers or are more time intensive or require creative set up.^9^,^10^ This nipple cover task trainer gives a realistic feel for anesthetizing and incising abscesses using affordable material and requires minimal preparation time. Even centers with limited simulation capabilities can create and use this task trainer because it uses material that is readily available.

**Educational Objectives:**

By the end of this training session, learners will be able to anesthetize an abscess, perform incision and drainage, develop manual dexterity maneuvering instruments to break up the abscess, and place packing using both the linear incision and loop techniques.

**Educational Methods:**

The abscess task trainers were fabricated using pre-made nipple covers, plastic wrap, and unscented hand lotion. The nipple covers come with a sticky backside that can allow adherence to plastic wrap. The plastic wrap is then filled with hand lotion and folded to prevent leakage. The nipple covers can then be anesthetized and incised. The time to fabricate each abscess was approximately one to two minutes.

**Research Methods:**

Eight PGY-1 emergency medicine residents completed a pre-simulation survey evaluating their confidence in draining an abscess using a five-point Likert scale (1=strongly disagree, 5 = strongly agree). Residents observed the instructor demonstrate the procedure, and then they performed two abscess drainages on separate nipple covers, one using a single linear incision and the other the loop technique. After the simulation, the resident confidence levels were reassessed using the same five-point Likert scale. Residents were also asked to rate the fidelity of the task trainer, compared to a real abscess (1= strongly disagree, 5 = strongly agree).

**Results:**

Residents reported an increase in their mean confidence in draining an abscess, with an increase from the pre-simulation score of 3.5 to 4.875 (p=0.0038). Residents also felt that the model was realistic, with a mean score of 4.875. Every resident recommended using this model for future learners.

**Discussion:**

Overall, this affordable and simple task trainer was well received by the learners and improved beginner confidence with a frequently performed procedure. With minimal preparation time and resources, this nipple cover task trainer can be used to teach residents how to anesthetize, incise, drain, and pack abscesses.

**Topics:**

Abscess, incision and drainage, simulation, task trainer.

## USER GUIDE

**Table:** 

List of Resources:	
Abstract	1
User Guide	3

**Learner Audience:**

Medical Students, Interns

**Time Required for Implementation:**

Preparation: Approximately one to two minutes per model for creation time ahead of the simulation session. Ideally, create two to three nipple pads per resident.

Didactics: A 30-minute simulation or didactic session is ideal for this task trainer. The instructor should spend 10 minutes demonstrating the proper technique for incision and drainage including linear incision and loop techniques.

Learners will need an additional 10 minutes to perform each technique, with a total of 30 minutes for the full session from start to finish.

**Recommended Number of Learners per Instructor:**

The ratio of learners to instructors should not exceed six to one.

**Topics:**

Abscess, incision and drainage, simulation, task trainer.

**Objectives:**

By the end of this training session, learners will be able to:

Anesthetize an abscess.
Perform incision and drainage using two recognized techniques.
Develop manual dexterity maneuvering instruments to break up the abscess.
4. Place packing or a loop in the abscess.

### Linked objectives, methods and results

Early learners are not formally taught how to incise and drain an abscess prior to performing the procedure in the clinical setting. This simulation session utilizes a low cost and high-fidelity task trainer to teach learners how to perform the procedure using two different techniques. The didactic session includes a detailed explanation by the instructor on how to perform the I&D procedure (objectives 1 and 2), after which learners participate in a hands-on session with the task trainers (objective 3 and 4). This allows learners to observe the best practice methods prior to performing the procedures themselves.

### Recommended pre-reading for instructor

Review your incision and drainage techniques for standard management of abscesses. Consider standard reference materials such as Roberts and Hedges, and these articles:

- How to incise and drain an abscess. Merck Manual Professional Edition, Merck & Co., Inc.^4^
- The loop technique for abscess management, PMID: 31663897.^6^

### Implementation Methods

Nipple covers should be prepared prior to the simulation session. For easy clean up, towels or a plastic tablecloth can be placed on the table underneath the task trainers. Two nipple cover task trainers should be given to each resident. Each resident should be provided gloves, a scalpel, forceps, ¼ or ½ cm gauze packing, and something to use as a loop drain. Traditionally a vascular loop is used in the clinical setting,^5^,^6^ but a quick and simple loop tie can be created by cutting an IV tourniquet in half lengthwise and widthwise or snipping open a long rubber band circle. The instructor should first demonstrate the two I&D techniques. Injecting lidocaine into the task trainer is not recommended, but the needle can be inserted to demonstrate the direction in which an anesthetizing agent should be applied. Using one nipple cover, residents can anesthetize and perform the traditional abscess drainage technique by cutting a linear incision along the length of the abscess, breaking up loculations using forceps, and then placing packing into the abscess. With the other nipple cover, residents can practice an incision and loop drainage by cutting two parallel 1 cm incisions in the covers and sliding a rubber loop through the two incisions and tying a circle. The abscess material drains very realistically compared to a real abscess. These task trainers are single use only.

### List of items required to replicate this innovation

1. Reusable silicone nipple covers pack of 12 (Amazon) $7.99
2. Fragrance free daily lotion (Amazon) $12.99
3. Plastic wrap $3.99
4. 25 or 27-gauge needles for anesthetizing.
5. 10 cc syringe
6. Scalpel blades
7. Forceps
8. ¼ or ½ cm gauze packing
9. Rubber loop – strips cut from rubber IV tourniquet or rubber bands.

### Options for purchasing

1. https://www.amazon.com/Nunibum-Nipple-Covers-Silicone-Pasties/dp/B0B4WK339F
2. https://www.amazon.com/Lubriderm-Moisture-Lotion-Fragrance-Free-Normal/dp/B07957S7QK

### Approximate cost of items to create this innovation

The nipple pads used were $8.00 for a package of 12. Any unscented lotion or barrier cream can be used for the abscess material. Plastic wrap is easily purchased from the store. The cost was around $1.25 each per I&D task trainer.

### Detailed methods to construct this innovation

1. Take the nipple covers out of packing. The shape does not matter for abscess.
  **Figure:**
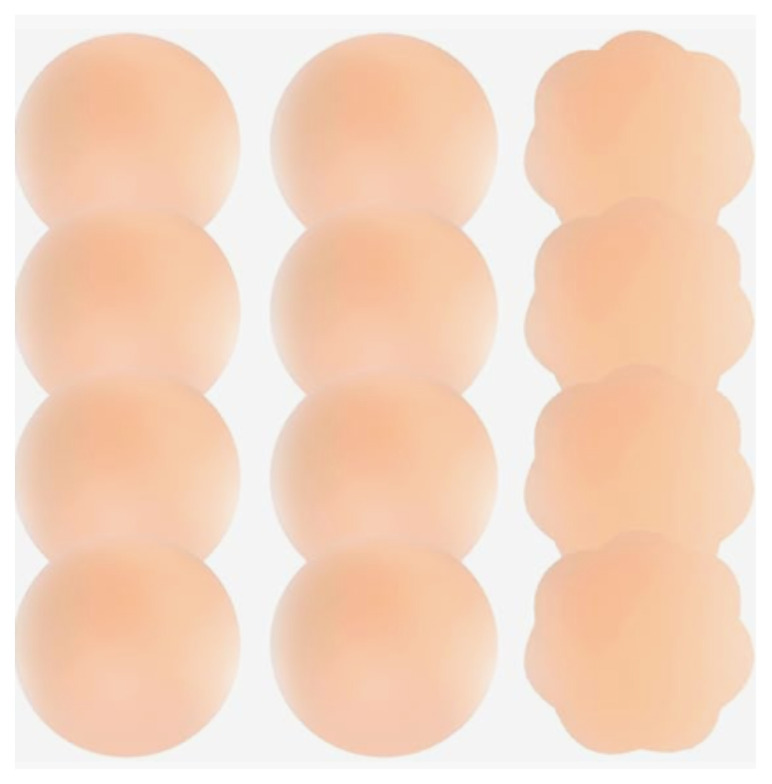
2. Cut a square piece of plastic wrap
3. Take the paper off the back of the adhesive silicone nipple cover
4. Stick the nipple cover directly to the plastic wrap.
  **Figure:**
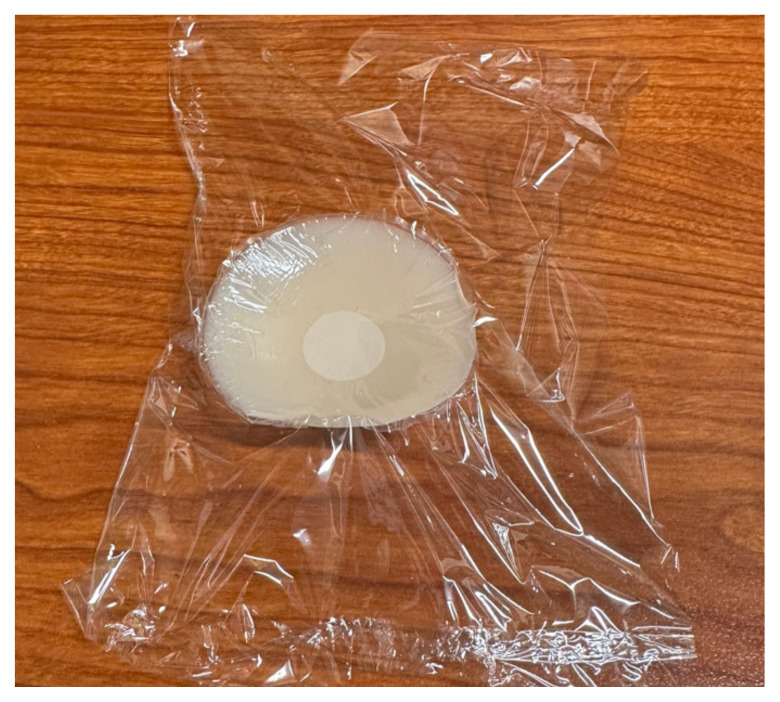
5. Fill back of nipple cover with ample amount of lotion (about a fourth cup to half a cup of lotion per abscess.)
  **Figure:**
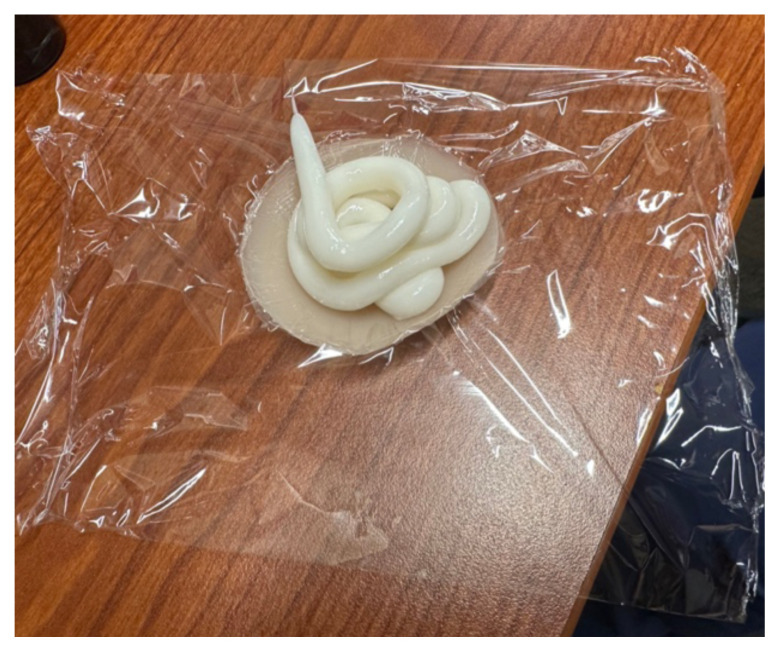
6. Wrap plastic wrap around the lotion to avoid any leakage.
  **Figure:**
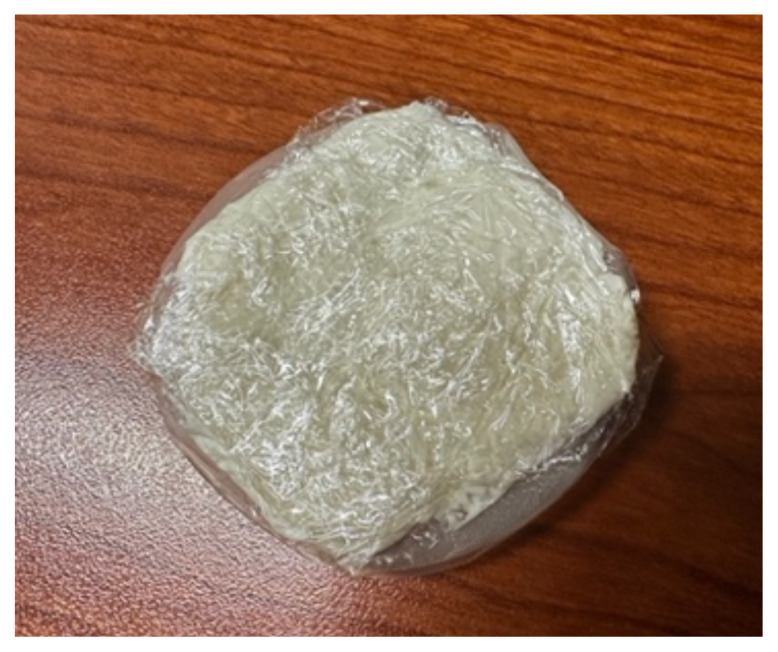
7. Optional: Create abscess incision and drainage kits with needle and syringe, scalpel, forceps, gauze packing and loop drain. Either an IV tourniquet or rubber band can be used for the loop drain.
  **Figure:**
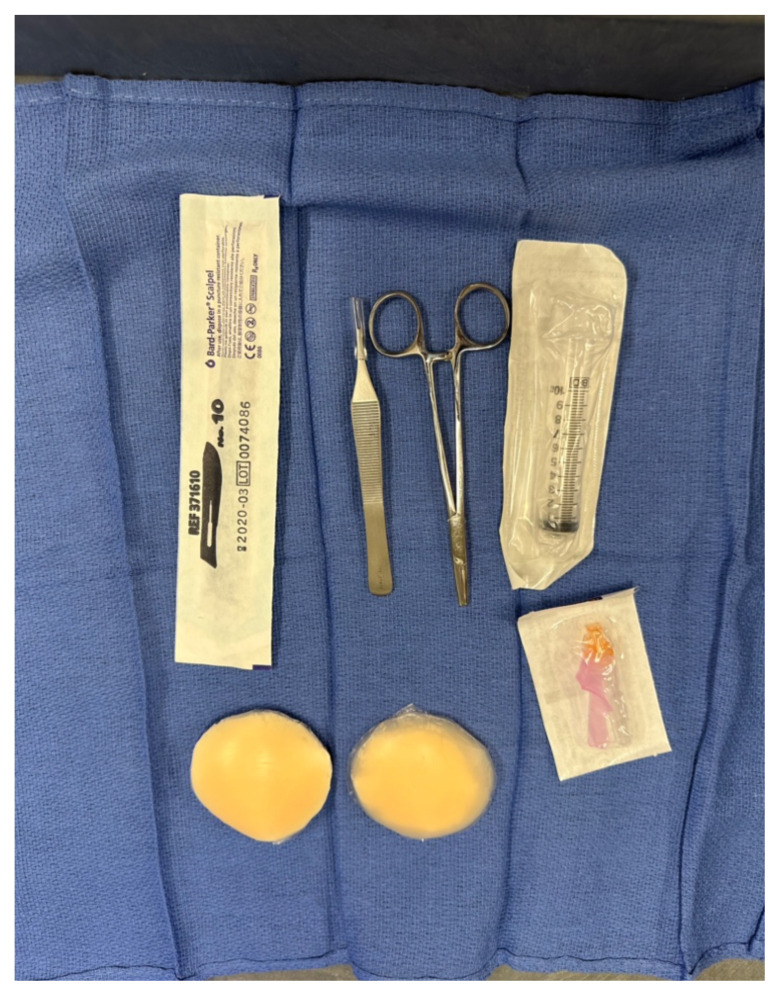

**Figure:**
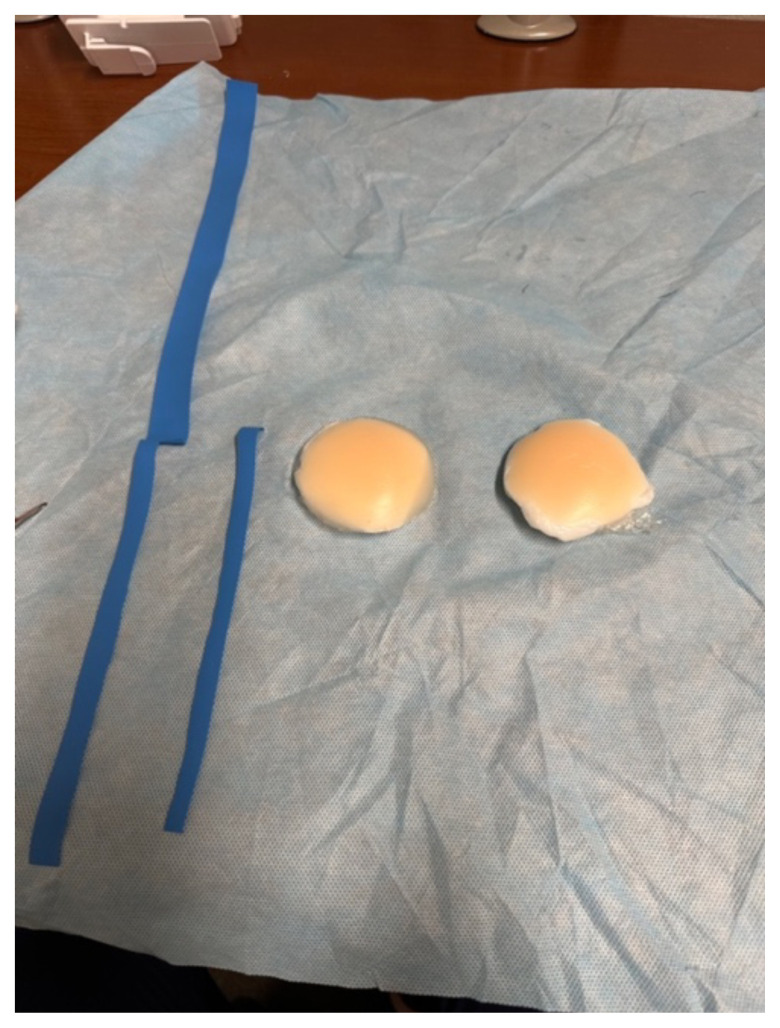

### Results and tips for successful implementation

**Figure 1:**
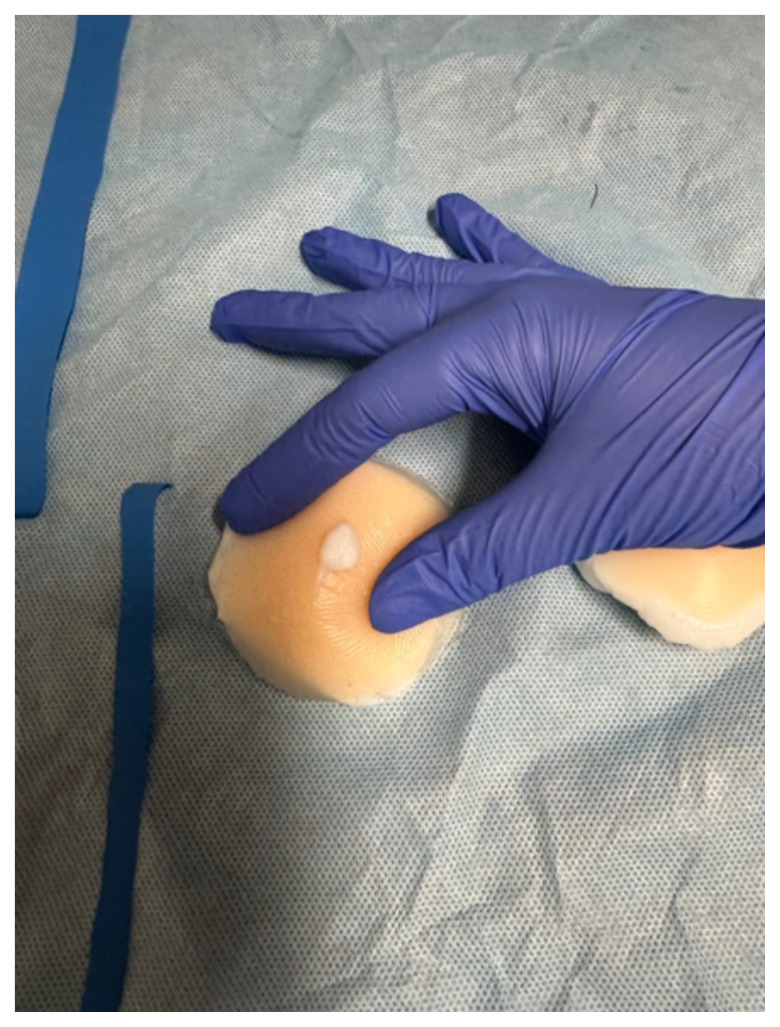
Incised abscesses with loop and packing placed and picture demonstrating “purulent” drainage.

Prior to performing two I&D’s, eight PGY-1 emergency medicine residents were asked if they were confident in draining a real abscess using a five-point Likert scale (1=strongly disagree, 5 = strongly agree). Residents were then given two nipple covers each. On one nipple cover, they were instructed on how to anesthetize then drain the abscess by a single linear incision. After the instruction, residents then incised one nipple cover, broke up loculations with forceps and inserted gauze packing. They repeated the I&D procedure on the second nipple cover; however, this time performing the loop technique (Figure 1). After the simulation, the resident confidence levels with draining an abscess were reassessed using the same five-point Likert scale. The means of the pre- and post-simulation confidence levels were compared using a paired T test calculator. Residents were also asked to rate the fidelity of the task trainer, compared to a real abscess (1= strongly disagree, 5 = strongly agree).

All eight participants completed the surveys. Two (33.3%) of the participants were female, and six were male (66.7%). When comparing pre-and simulation confidence, there was an increase in their mean confidence from the pre-simulation score of 3.5 to 4.875 (p=0.0038). Residents also felt that the model was realistic, with a mean score of 4.875. Every resident agreed or strongly agreed that they should be used for future learners. Residents appreciated the simple task trainer including written feedback: “This was great and so easy to use!”

**Figure:**
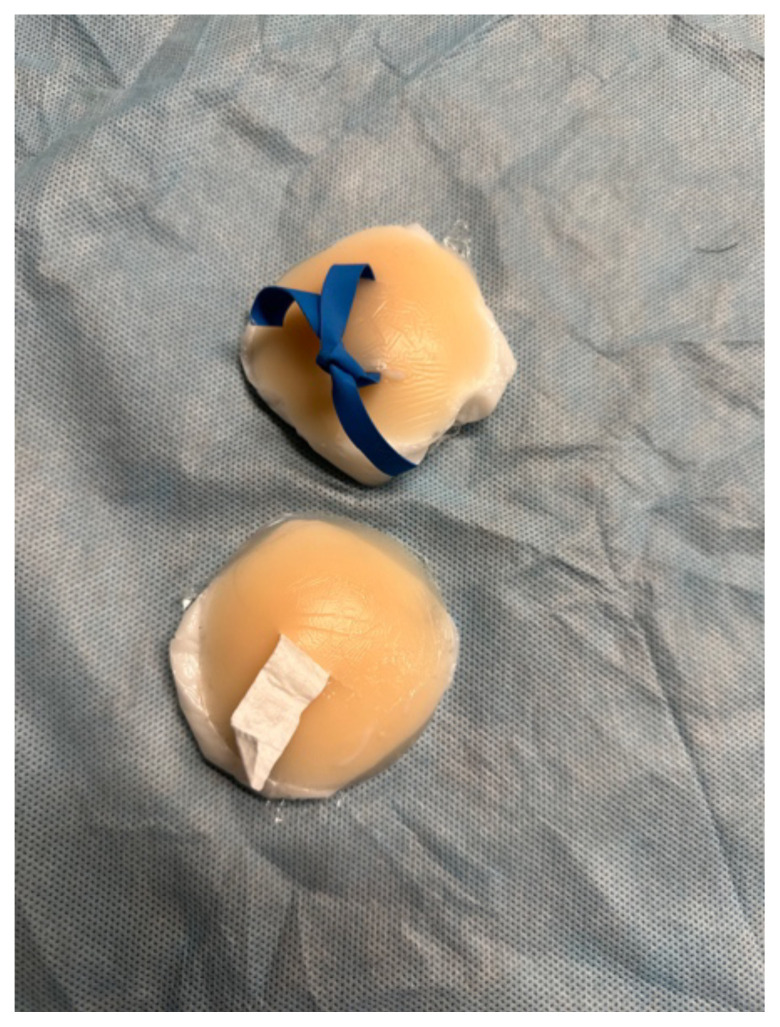


### Discussion

This affordable and simple task trainer was well received by the learners and improved beginner confidence with a frequently performed, but not often simulated procedure. To create the nipple cover task trainer, minimal preparation time and resources are required, making this a simulation that can be replicated in even resource-sparse residency or medical student programs. In the future, comparing this task trainer with a commercially available task trainer would be beneficial to improve task trainer fidelity.
